# Metabolic Engineering of *Saccharomyces cerevisiae* for Caffeine and Theobromine Production

**DOI:** 10.1371/journal.pone.0105368

**Published:** 2014-08-18

**Authors:** Lu Jin, Mohammad Wadud Bhuiya, Mengmeng Li, XiangQi Liu, Jixiang Han, WeiWei Deng, Min Wang, Oliver Yu, Zhengzhu Zhang

**Affiliations:** 1 Key Laboratory of Tea Biochemistry and Biotechnology, Ministry of Education, Anhui Agricultural University, Hefei, PR China; 2 Conagen Inc., St. Louis, Missouri, United States of America; 3 Donald Danforth Plant Science Center, St. Louis, Missouri, United States of America; University of Geneva, Switzerland

## Abstract

Caffeine (1, 3, 7-trimethylxanthine) and theobromine (3, 7-dimethylxanthine) are the major purine alkaloids in plants, e.g. tea (*Camellia sinensis*) and coffee (*Coffea arabica*). Caffeine is a major component of coffee and is used widely in food and beverage industries. Most of the enzymes involved in the caffeine biosynthetic pathway have been reported previously. Here, we demonstrated the biosynthesis of caffeine (0.38 mg/L) by co-expression of *Coffea arabica* xanthosine methyltransferase (CaXMT) and *Camellia sinensis* caffeine synthase (TCS) in *Saccharomyces cerevisiae*. Furthermore, we endeavored to develop this production platform for making other purine-based alkaloids. To increase the catalytic activity of TCS in an effort to increase theobromine production, we identified four amino acid residues based on structural analyses of 3D-model of TCS. Two TCS1 mutants (Val317Met and Phe217Trp) slightly increased in theobromine accumulation and simultaneously decreased in caffeine production. The application and further optimization of this biosynthetic platform are discussed.

## Introduction

**Figure 1:**
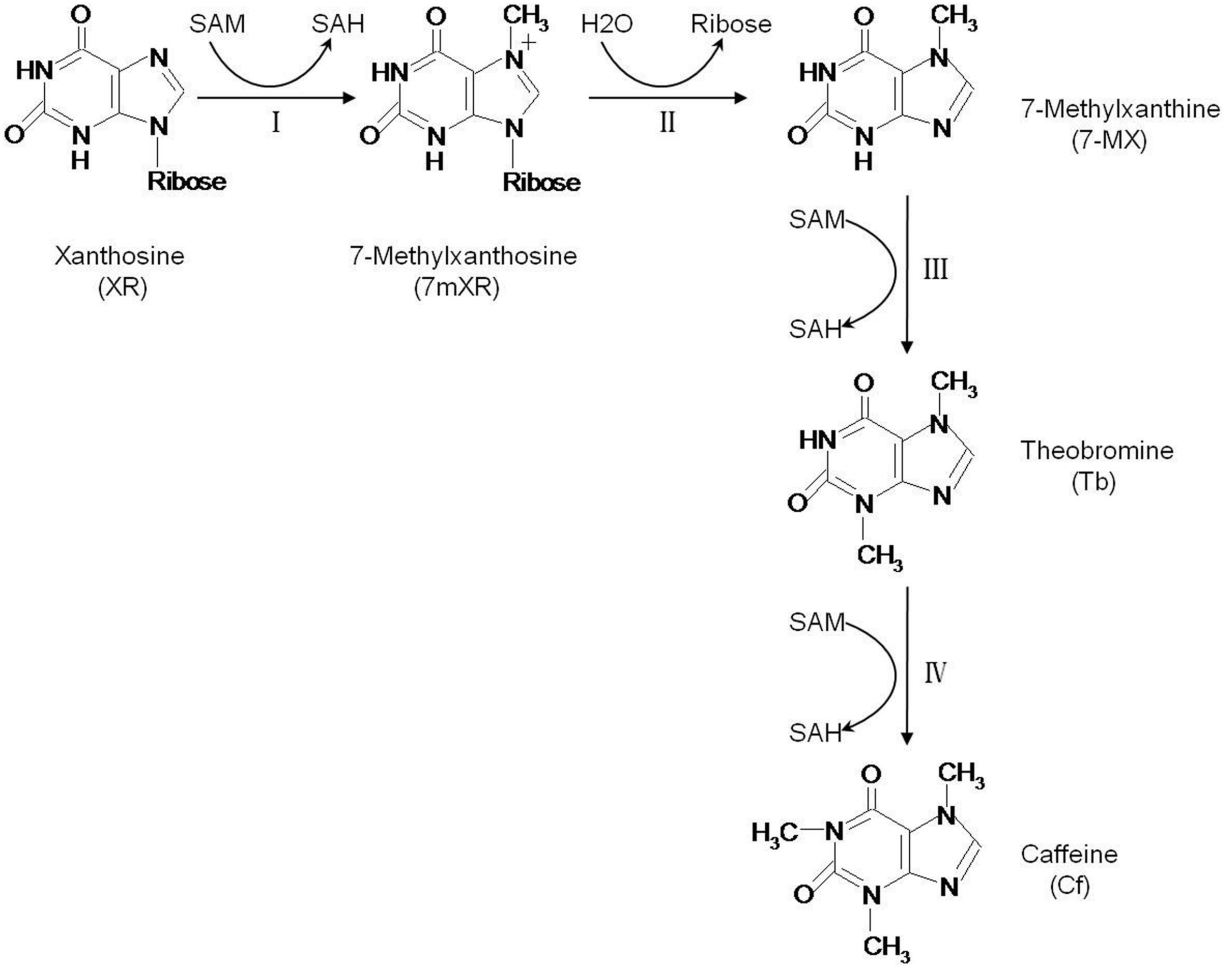
The four steps of the caffeine biosynthetic pathway in plants. Step I, the conversion of xanthosine (XR) to 7-methylxanthosine (7-mXR) by xanthosine methyltransferase (CaXMT1); Step II, the hydrolysis of 7-mXR to 7-methylxanthine (7-MX) by N-methyl-nucleosidase; Step III, the methylation of 7-MX to Theboromine (Tb) by caffeine synthase (TCS1); Step IV, the methylation of Tb to caffeine (Cf) by caffeine synthase (TCS1).

Caffeine (1, 3, 7-trimethylxanthine) and theobromine (3, 7-dimethylxanthine) are the major purine alkaloids in plants, e.g. tea (*Camellia sinensis*) and coffee (*Coffea arabica*), and the metabolic engineering of alkaloid pathways has been reported [1]–[7]. The biosynthetic pathway of caffeine require four steps [8]–[11], including three methylation reactions catalyzed by *N*-methyl-transferases and one nucleoside cleavage reaction catalyzed by nucleosidase (Fig. 1). The four steps of the caffeine biosynthetic pathway in plants are Step I, the conversion of xanthosine (XR) to 7-methylxanthosine (7-mXR) by xanthosine methyltransferase (CaXMT1); Step II, the hydrolysis of 7-mXR to 7-methylxanthine (7-MX) by N-methyl-nucleosidase; Step III, the methylation of 7-MX to Theboromine (Tb) by caffeine synthase (TCS1) and Step IV, the methylation of Tb to caffeine (Cf) by caffeine synthase (TCS1).

*S-*adenosyl-*L*-methionine (SAM) is the methyl donor for *N*-methyltransferases (NMTs) in caffeine biosynthesis [12]. Most of the methyltransferase enzymes involved in caffeine biosynthetic enzymes have been reported [8], [10], [13]–[15]. Radioactive tracer analysis identified XR, an intermediate of the adenine and guanine pathway, as the immediate precursor of caffeine. The CaXMT1 catalyzes 7-N-methylation of XR to form 7-mXR. Isolation, identification and expression of CaXMT1 from *Coffea arabica* was reported [16]. The *N*-methyl-nucleosidase from *C. arabica* catalyzes the hydrolysis of 7-mXR to form 7-MX after removal ribose [17]. The 7-methylxanthosine synthase from *C. arabica* showed dual activity and catalyzes both methylation and hydrolysis for conversion of XR to 7-MX [18].

The caffeine synthase (TCS1) from *Camellia sinensis* catalyzes 3-N methylation of 7- MX to form Tb and subsequently 1-N methylation of Tb to form Cf [11], [19]. Caffeine synthase (*C. arabica*) and theobromine synthases (*C. arabica and Theobroma cacao*) showed dual methyltransferase and mono methyltransferase activity, respectively [13], [16], [20]–[21]. Co-expression of caffeine or thebromine synthases with 7-methylxanthosine synthase can be used for production of both Cf and Tb. Here, we demonstrated the caffeine biosynthesis pathway by co-expression CaXMT1 and TCS1 in *Saccharomyces cerevisiae*. Since the amount of caffeine and theobromine produced by this pathway is low, structure-guided mutagenesis of TCS1 has been carried out for increasing the production of caffeine.

## Materials and Methods

### Plant material, RNA isolation and cDNA amplification

Coffee (*C. arabica*) and tea (*C. sinensis*) plants (1∼2 years old) were grown at the Yunnan Plant Science Center and the Anhui Agricultural University Experimental Tea Garden, respectively. For cDNA synthesis, fresh tea shoots were harvested and stored at −80°C until use.

Total RNA was extracted using the RNeasy Plant Mini Kit (Qiagen, Madison, WI, USA) and converted into single-strand cDNA using PrimeScript 1st Strand cDNA Synthesis Kit (Takara, Oshu, Japan). DNA sequence analysis and translation were done using Vector NTI Advance 11.5 (Invitrogen, Pasadena, CA, USA).

### Construction of CaXMT1 and TCS1 vectors for over-expression in *E. coli* Rosetta (BL21, DE3)

The forward primers CaXMT-F1 and TCS-F1 (Table 1) both contained a starting codon and a *Bam*HI restriction site, while the reverse primers CaXMT-R1 and TCS-R1 (Table 1) contained *Sal*I. PCR reactions were performed using PrimeSTAR HS DNA polymerase (Takara) under the following PCR conditions: Denaturation at 98°C for 10 s, annealing at 60°C for 15 s, and extension at 72°C for 1 min, repeated for 30 cycles. The PCR products were sub-cloned into pMD18-T Simple vector (Takara). Nucleotide sequencing was carried out to confirm the resulting plasmids (Invitrogen Pasadena, CA, USA). The excised *Bam*HI-*Sal*I fragments of CaXMT1 and TCS1 were cloned into the GST fusion vector pGEX-4t-2 at the corresponding sites (TransGene Biotech, Beijing, China). The resulting plasmids were transformed into the expression host *E. coli* Rosetta (BL21, DE3) by heat shock. Empty vector was also transformed as a control.

**Table 1:** Oligonucleoside primers for PCR.

Name	Sequence	Used for
CaXMT1-F1	5′-GCGGATCCATGGAGCTCCAAGAAGTCCTG-3′	Cloning and expression in *E. coli* as GST fusion
CaXMT1-R1	5′-CCGGTCGACCACGTCTGACTTCTCTGGCT-3′	
TCS1-F1	5′-CTCGGATCCATGGAGCTAGCTACTGCGGG-3′	
TCS1-R1	5′-CCGGTCGACTCCATCAATCTTGGAAAGCA-3′	
ST1-For	5′-CTCGGATCCATGGAAACCTCAGCTGCTAA-3′	
ST1-Rev	5′-CCGGTCGACAGCACTTGAAATTATGTAAT-3′	
ST2-For	5′-CTCGGATCCATGCTGAACATAGTAATATT-3′	
ST2-Rev	5′-CCGGTCGACGTGGTCTTCTAGAGTTTGAT-3′	
ST3-For	5′-CTCGGATCCATGATGAATGCAGCTGCAAC-3′	
ST3-Rev	5′-CCGGTCGACGAATTGGGAAGGCATTTTAT-3′	
ST1-FY	5′-CACCATGGAAACCTCAGCTGCT-3′	Cloning and expression in *S. cerevisiae*
ST1-RY	5′-CTAAGCACTTGAAATTATGTAATTG-3′	
ST2-FY	5′-CACCATGCTGAACATAGTAATATTAG-3′	
ST2-RY	5′-TCAGTGGTCTTCTAGAGTTTG-3′	
ST3-FY	5′-CACCATGATGAATGCAGCTGCAAC-3′	
ST3-RY	5′-TTAGAATTGGGAAGGCAT-3′	
TM1-F	5′-TGTATGGTGTTGATACTTCATGGTAGGCAATGT-3′	Site-directed mutagenesis
TM1-R	5′-ACCATTTGGAACCACCTCTTGGGATCTAGC-3′	
TM2-F	5′-GGGGAAAAGTTTACCAAGATGGTCAGGGCCTC-3′	
TM2-R	5′-TCTAACCCATTTATCATTCTCCTGCATTTC-3′	
TM3-F	5′-CAATATACCCAGCTATTGGGCATCACTTGAG-3′	
TM3-R	5′-AAGGTGTCTAATTTATCTTCATCTATCAATC-3′	
TM4-F	5′-CAATATACCCAGCTATTTTCCATCACTTGAGG-3′	
TM4-R	5′-AAGGTGTCTAATTTATCTTCATCTATCAATC-3′	
CaXMT1-R2	5′-CCGGTCGACCACGTCTGACTTCTCTGGCT-3′	Co-expression In *E. coli*
TCS1-F2	5′-GCGTCGACATGGAGCTAGCTACTGCGGG-3′	
TCS1-R2	5′-TTGCGGCCGCCTATCCATCAATCTTGG-3′	

### Growth conditions and solubility measurement

For *in vivo E. coli* assays [16], a single colony growing on selective media was cultured at 37°C over-night in 3 mL of Luria Broth containing 0.1 mg/mL ampicillin with constant shaking (200 rpm). Aliquots (500 µL) of the bacterial culture were added into 25 mL of fresh LA and incubated at 37°C for 2 h with shaking. Expression of GST-fusion proteins was induced by adding 25 µL of 0.5 M isopropyl-β-D-thinogalactoside (IPTG, final concentration 0.5 mM), and cell growth was continued for 4 h at 30°C. *E. coli* cells were harvest by centrifugation at 6000 rpm for 5 min, suspended in lysis buffer containing 50 mM Tris-HCl (pH 8.0), 1 mM EDTA and 5 mM dithiothreitol (DTT), sonicated to disrupt cells and then centrifuged. The supernatant was used as a crude enzyme mix in the assay for caffeine metabolism. Protein concentration was determined by UV spectrophotometer. Ten micrograms of each sample were subjected to SDS-PAGE analysis on a 10% (w/v) gel and visualized by Coomassie Brilliant Blue staining.

### Enzymatic activity assays *in vitro* and single functional analysis by HPLC and LC-MS

For *in vitro* analysis, reactions with crude GST-fusion protein extracts containing 200 mM MgCl_2_, 100 µg/mL of substrates (XR and 7-MX, Sigma-Aldrich, St Louis, MO, USA), and 50 µg/mL SAM were incubated at 30°C for 8 h. The samples were centrifuged. The supernatants were passed through a 0.45 µM filter and analyzed by HPLC using a Phenomenex reverse-phase C18 column (5 µm, 4.6×250 mm). Solvent A was 0.2% acetic acid while B was 100% acetonitrile. The linear gradient was as follows: at 0 min, 92% A; at 25 min, 83% A; at 30 min, 10% A; at 32 min, 10% A; and finally from 35 to 40 min, 92% A. The flow rate was 1 mL/min and injection volume was 10 µL. Mass spectrometry conditions were as follows: EPI ion source, 40 arb of sheath gas velocity, 10 arb of auxiliary gas velocity, 3.5 kV of spray voltage, 150°C capillary temperature, 45 V capillary voltage, 30 V tube lens shift and 18 V energy.

### Construction of CaXMT1 and TCS1 vectors for over-expression in yeast S. *cerevisiae*

The ORFs of CaXMT1 and TCS1 were both commercially synthesized (DNA 2.0 Inc., Menlo Park, CA, USA). The amino acid translations of the synthesized DNA fragments were identical to the original ones. The nucleotides ‘ATCC’ were added before the start codon ‘ATG’ for convenient Gateway manipulation (Invitrogen, Pasadena, CA, USA). DNA fragments were cloned into the pENTR/D-TOPO vector and transformed into One Shot *E. coli* competent cells. After sequencing, the resulting genes were separately introduced into the Advanced Gateway destination yeast vectors pAG425GAL-ccdB (-LEU) and pAG424GAL-ccdB (-TRP) (Addgene, Boston, MA, USA) by LR Clonase II enzyme mix kit. The recombination reaction products pAG425GAL-CaXMT1 and pAG424GAL-TCS1 were co-transformed into yeast *S. cerevisiae* INVSC1 cells (Invitrogen, Pasadena, CA, USA) made competent by Frozen-EZ Yeast Transformation II kit (Zymo Research, Orange, CA, USA). Empty vector pAG425GAL-ccdB was also transformed as a control. Transformants were plated on solid SD (selective dropout) dropout selective medium (-TRP and -LEU) at 30°C for 2–3 days until colonies appeared.

### Growth conditions, co-expression in yeast *S. cerevisiae* and functional analysis

For *in vivo* yeast assays [22], a single colony transformed with pAG425GAL-CS1+ pAG424GAL-CS2 was inoculated into 3 mL of SD dropout selective medium (-LEU and -TRP) containing 2% (w/v) glucose and incubated at 30°C with shaking (250 rpm) for 24 h. A portion (2 mL) of the yeast cells was transferred to 20 mL of fresh SD dropout liquid medium with 2% raffinose for additional culture until the OD_600_ reached 1.0. Galactose was then added to a final concentration of 2% for induction. Meanwhile, 100 µM of xanthosine (XR; Sigma-Aldrich, USA) was fed as substrate. And no xanthosine feeding was also performed as control. After 24 h of galactose induction, the compounds were extracted from the culture media and yeast cells using ethyl acetate. The upper organic phase was evaporated under nitrogen using a pressurized gas blowing concentrator. A suspension was made with the addition of distilled water.

Aliquots of the above samples were analyzed on an Agilent 1100 series HPLC system using a Phenomenex reverse phase C18 column (3.5 µm; 4.6 mm×250 mm). Samples were diluted into acetic acid and then separated using a 25-min, linear gradient from 92% water and 8% acetonitrile to 70% water and 30% acetonitrile at a flow rate of 1.0 mL/min. The injection volume was 20 µL and the UV detection wavelength was 272 nm.

For LC-MS analysis, an Applied BioSystems QSTAR XL (Applied Biosystems, Foster City, CA, USA) hybrid quadrupole, multiple reactions monitoring (MRM) MS system equipped with a nano-electrospray source (Protana XYZ manipulator) was used to confirm the identities of the compounds in the yeast extract, following previous protocols [23].

### Screening, cloning and functional analysis of nucleosidase candidate genes

A tea transcriptome library was constructed from seven different tissues [24]. Based on assembly and functional prediction analysis, six genes were selected as candidate nucleosidase genes. Firstly, to obtain gene expression profiles, total RNA was isolated separately from the root and leaf of tea seedlings by RNeasy Plant Mini Kit (Qiagen). Contaminating DNAs were removed by TURBO DNA-free Kit (Ambion, Boston, MA, USA). Reverse transcription by ImPro-II Reverse Transcription System (Promega, Madison, WI, USA) was performed to obtain cDNA templates for semi-quantitative RT-PCR analysis. The primer pairs ST1-FY, ST1-RY, ST2-FY, ST2-RY, ST3-FY and ST3-RY are listed in Table 1. The 30 reaction cycles followed denaturation at 94°C for 30 s, annealing at 50°C for 30 s, and extension at 72°C for 1 min. After the amplified cDNAs were sub-cloned into the pENTR vector, the genes ST1, ST2 and ST3 were then transformed into yeast vector pAG-423GAL-ccdB (-His) by LR Clonase II enzyme mix kit (Invitrogen, Pasadena, CA, USA). Co-expression of each of the nucleosidases with pAG425GAL-CaXMT1 and functional analysis were carried out as previously described [23].

### Site-directed mutagenesis of tea caffeine synthase (TCS1)

Mutagenesis was performed at sites 225, 271, 272 and 317 of TCS1 following the QuickChange site-directed mutagenesis strategy (Stratagene, CA, USA). The primers used to construct mutants are listed in Table 1. PCR was performed using phusion high-fidelity DNA polymerase (Finnzymes, Espoo, Finland) with the reaction settings at 98°C for 50 s, 1 cycle, followed by 25 cycles at 98°C for 10 s, 60°C for 15 s, and 72°C for 6 min; the final extension was 72°C for 10 min. The QuikChange PCR products were examined by agarose gel electrophoresis and then 15 µl of PCR products were digested with 1 µl DpnI (New England Biolabs Beijing, China) at 37°C for 4 hrs to remove the template plasmid. Aliquots of (2 µl) digestive products were transformed into *E. coli* Rosetta (BL21, DE3) competent cells (Stratagene, CA, USA). The mutants were confirmed by DNA sequencing of the TCS1 plasmids. For the further duplication, a new expression vector with high efficiency, pMAL-c5X (NewEnglandBiolabs, Beijing, China) was used instead of pGEX-4t-2 (Transgen Biotech, Beijing, China). The resulting plasmids were transformed into the expression host *E. coli* Rosetta (BL21, DE3). For *in vitro* analysis, reactions with crude fusion protein extracts containing 200 mM MgCl2, 1140 µM 7-MX and 300 µM SAM were incubated at 25°C for 5 h. After reaction, samples were determined by HPLC. In this improvement, at least four independent duplications were performed.

### Co-expression of CaXMT1 and TCS1 in *E. coli* Rosetta (BL21, DE3) system

For *E. coli* expression with the GST-fusion, the coding regions of CaXMT1 and TCS1 were re-cloned using the previous primer Ca-For primer and new primers: Ca-New-Rev, TC-New-For, and TC-New-Rev (Table 1). The PCR products were first sub-cloned into pMD18-T Simple vector (Takara). The sub-clone of CaXMT1 was digested with *Bam*HI and *Sal*I and of TCS1 with *Sal*I and *Not*I. The *Bam*HI*/Sal*I and *Sal*I/*Not*I fragments were both introduced into the GST fusion vector pGEX-4t-2 (TransGene Biotech, Beijing, China) at the *Bam*HI and *Not*I sites. The *in vivo* and *in vitro* production of caffeine and related compounds were analyzed as above. For the further improvement, a new high efficiency expression vector, pMAL-c5X (NewEnglandBiolabs, Beijing, China) was used instead of pGEX-4t-2 (Transgen Biotech, Beijing, China) afterwards. At least four independent duplications were performed.

### Homology modeling

The amino-acid sequences of TCS1 and ICS1 were aligned to that of *C. canephora* 1,7-dimethylxanthine methyltransferase (DXMT) using the CLUSTAL W version 1.83. The homology models of TCS1 and ICS1 were built based on the structure *C. canephora* DXMT (Protein Data Bank code 2EFJ) using the program I-tasser [25]. The substrate theobromine was docked into the built TCS1 model via the program SWISDOCK [26].

## Results

### Cloning and functional analysis of key enzymes involved in caffeine biosynthesis

**Figure 2:**
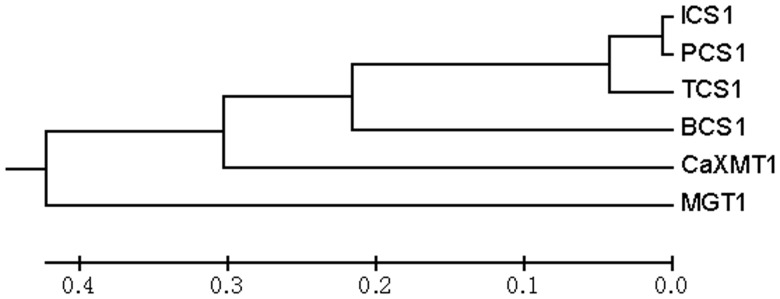
Phylogenetic tree of *N*-methyltransferases related to caffeine biosynthesis. Genes, accession numbers, species nomenclature, and substrates are listed in that order as follows: MGT1, X60368, *Saccharomyces cerevisiae*, unknown; CaXMT1, AB048793, coffee, xanthosine; TCS1, AB031280, tea, 7-methylxanthine and theobromine; ICS1, AB056108, *Camellia irrawadiensis*, 7-methylxanthine; PCS1, AB207817, *Camellia ptilophylla*, 7-methylxanthine; BCS1, AB096699, *Theobroma cacao*, 7-methylxanthine. The sequences were compared and phylogenetic tree was generated by MEGA5 (Tamura, Peterson, Stecher, Nei, and Kumar 2011). The branch lengths represent numbers of substituted residues per site.

For construction of caffeine biosynthetic pathway in microbial systems, we cloned the cDNA encoding CaXMT1 (AB048793) from coffee and TCS1 (AB031280) from tea (Fig. 2). The CaXMT1 fragment is 1316 bp in length and contained a single ORF of 1119 bp predicted to encode a 7-methylxanthosine synthase (EC 2.1.1.158). The peptide was predicted to contain 372 amino acid residues with an apparent molecular mass of 41.8 kDa. The TCS1 fragment was 1438 bp in length and contained a single ORF of 1110 bp predicted to encode a caffeine synthase (EC 2.1.1.160). This enzyme was predicted to carry out the last two steps of methyltransferase for caffeine biosynthesis, i.e. the conversion of 7-methylxanthine (XR) to caffeine via theobromine. The peptide consists of 369 amino acid residues with an apparent molecular mass of 41.3 kDa.

**Figure 3:**
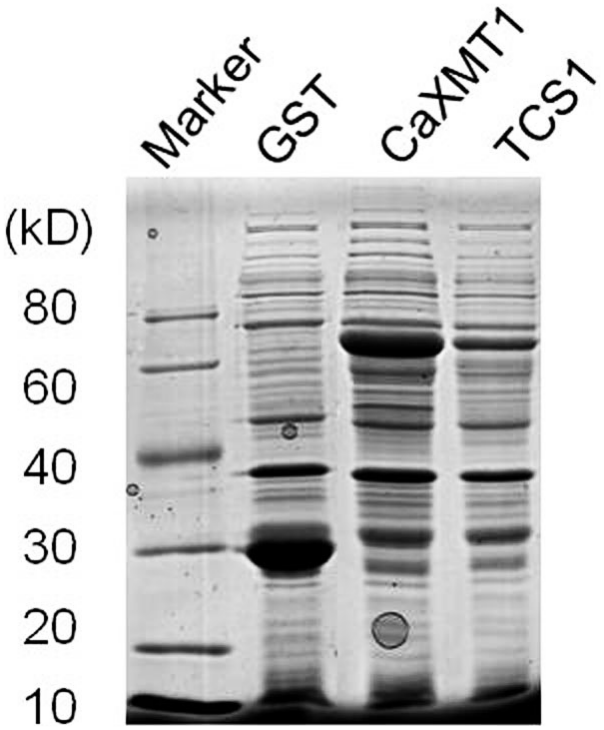
SDS-PAGE analysis of recombinant proteins showing the expression of caffeine biosynthetic enzymes. GST and the CaXMT1- and TCS1-GST fusion proteins were expressed in *E. coli* and prepared for gel loading. The samples were separated on a 10% (w/v) SDS-polyacrylamide gel and visualized by Coomassie Brilliant Blue staining. Arrows indicate recombinant proteins.

**Figure 4:**
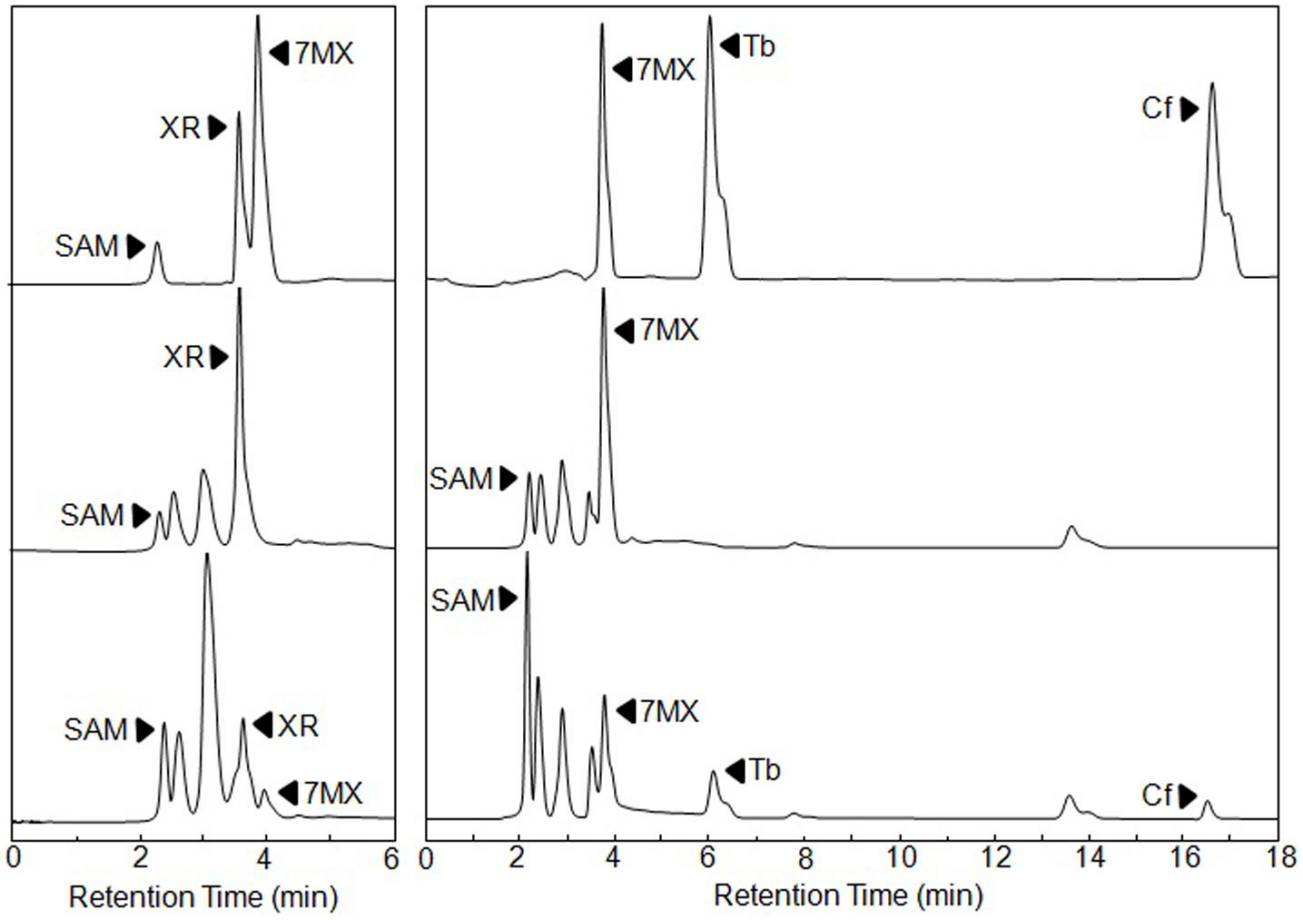
*In vitro* functional analysis of CaXMT1 and TCS1 expressed in *E. coli.* Extracts from *E. coli* expressing GST-CaXMT1 (a) and -TCS1 (b) were extracted and used for enzymatic assays. The top traces of (a) and (b) show the authentic standards run in parallel. The middle traces are negative controls. The bottom trace of (a) shows that CaXMT1 recombinant protein catalyzed the reaction from xanthosine (XR) to 7-methylxanthine (7-MX). The bottom trace of (b) presents the TCS1 catalysis of 7-MX to caffeine (Cf) via theobromine (Tb). Black arrowheads indicate reaction products.

We separately expressed these two NMTs in *E. coli* as GST-fusion proteins. The soluble proteins were detected in *E. coli* protein extracts (Fig. 3) and collected for methylation assays as a crude extract. The extract containing CaXMT1 was incubated with SAM and XR for 8 h, after which it was filtered to remove the proteins and directly analyzed by HPLC (Fig. 4a). In the enzyme assay section, negative controls (hosts with blank vector) were included as well. The production of 7-MX was detected, while the peak of the substrate XR was significantly reduced. LC-MS analysis confirmed the identity of the product (data not shown).

The reaction mixture contained crude extract of TCS, SAM and 7-MX and incubated for 8 h, after which the products were analyzed by HPLC (Fig. 4b). The production of theobromine and caffeine was detected. The amount of substrate reduced significantly. The LC-MS analysis confirmed the product (data not shown). Therefore, both GST-fusions of CaXMT1 and TCS were functional and presented enzymatic activities consistent with previous reports.

### Co-expression of CaXMT1 and TCS1 lead to caffeine biosynthesis in yeast

For construction of caffeine biosynthesis pathway in yeast, the two genes were sub-cloned into pAG-GAL yeast expression vectors from Addgene. The non-integrative plasmid vectors were transformed into yeast *S. cerevisiae* INVSC1 host cells independently. To confirm the function of each enzyme in yeast, the crude extract of yeast carrying pAG-GAL-CaXMT1 or pAG-GAL-TCS1 were prepared for *in vitro* enzymatic assays and HPLC analysis as above. The results indicated that CaXMT1 catalyzed the conversion of XR to 7-MX (data not shown) and that TCS1 catalyzed conversion of 7-MX to caffeine when expressed in *S. cerevisiae* (data not shown).

**Figure 5:**
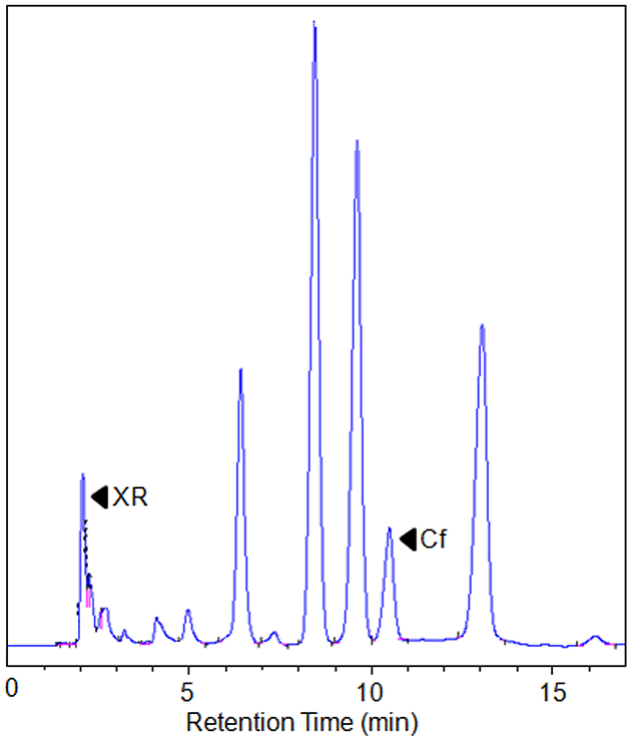
*In vivo* functional analysis of co-expression of CaXMT1 and TCS1 in *S. cerevisiae*. The HPLC trace shows that co-expression of CaXMT1 and TCS1 can catalyze the conversion from xanthosine (XR) to caffeine (Cf). Black arrowheads indicate reaction products.

The two plasmids were then co-transformed into yeast *S. cerevisiae* INVSC1. The transgenic yeast showed normal growth and development in minimal media containing glucose. Cells were grown for 12 hr, and then galactose was added to the culture media to induce expression. The entire culture, including the growing yeast cells, was extracted with ethyl acetate. The organic phase of the sample was dried under nitrogen, re-suspended with methanol, and subject to HPLC analysis. In the enzyme assay section, negative controls (hosts with blank vector) were included as well. When 100 µM XR was fed into the culture media as a substrate at the time of induction, caffeine was detected in strains co-transformed with pAG425GAL-CaXMT1and pAG424GAL-TCS1 (Fig. 5). An empty vector did not produce any caffeine. The yield of caffeine (caffeine content calculated from HPLC result) was 0.38±0.03 mg/L, suggesting that the conversion rate from fed xanthosine to caffeine was approximately 1.95% (the conversion rate defined as the ratio of actually produced caffeine to the theoretically synthesized caffeine from XR-feeding). Caffeine was not detected when exogenous xanthosine was not added.

Cells were separated by centrifugation and the caffeine was extracted independently. The majority of the caffeine was detected in the culture media. No caffeine was detected from the cells suggesting that there was no net directional transport of caffeine. To increase the extraction recovery rate, different solvents were tested. Ethyl acetate was a good solvent for caffeine extraction, about 12-fold more than dichloromethane, and was used for all other experiments.

### Cloning and co-expression of putative tea nucleosidase genes

Since the accumulation of caffeine was relatively low, we used various nucleosidase to increase caffeine accumulation. The catalytic efficiency of TCS1 (Step II) is two orders of magnitude higher than CaXMT1 (Step I) [27]. Therefore, we sought to remove this bottleneck by expressing a nucleosidase specific for converting 7-methylxanthosine (7-mXR) to 7-methyxanthine (7-MX) instead of relying on CaXMT1’s nucleosidase activity, which showed relatively low catalytic activity based on kcat/Km [16].

Endogenous nucleosidases from a variety of species often have broad substrate range but low catalytic efficiency [28]–[31]. These enzymes have similar kcat/Km values toward the substrates inosine, xanthosine, uridine, guanine, and adenine, ranging from 1.1 to 4.3×10^4^ kcat/Km. This suggested that general nucleosidases are inefficient enzymes. Furthermore, the functional characterization of bacterial nucleosidases from *Corynebacterium ammoniagenes*, nucleoside hydrolases (NHs), showed that their substrate range and catalytic efficiencies were similar to previous reports [32].

Taken together, we hypothesized that CaXMT1 or the yeast endogenous nucleosidase could remove the ribose residue from 7-mXR to form 7-MX, but a more specific nucleosidase from caffeine-producing plants may significantly increase the pathway efficiency.

**Figure 6:**
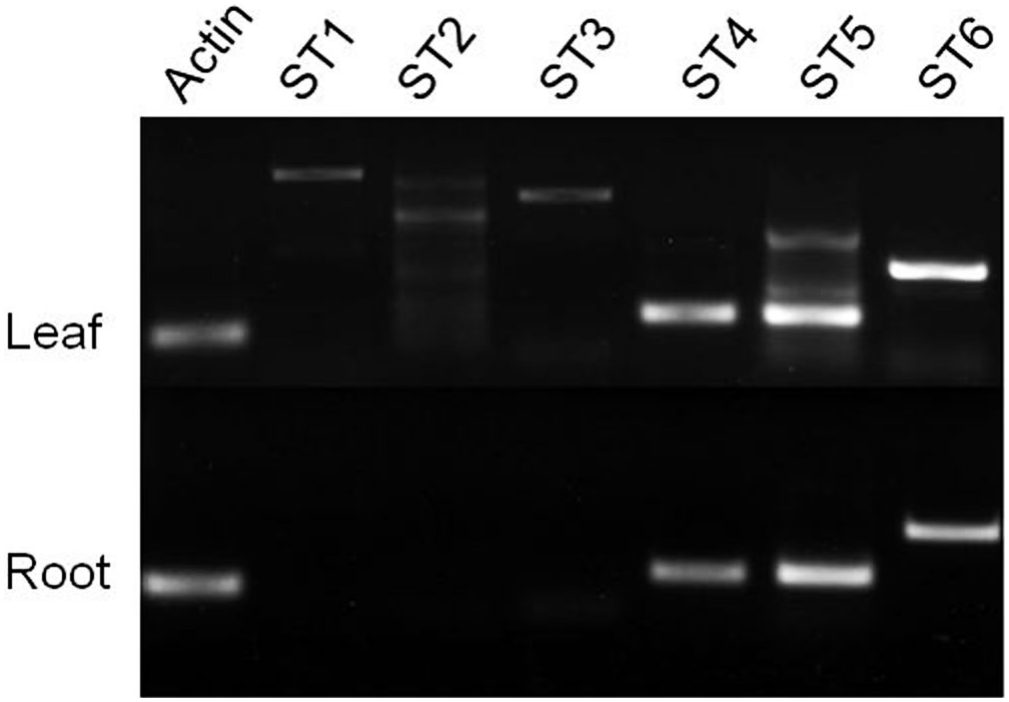
Semi-quantitative RT-PCR amplification of putative nucleosidase genes. Total RNA samples were prepared separately from different tissues of tea. Actin gene was used as the internal standard. Semi-quantitative RT-PCR analysis was performed using the gene-specific primer sets listed in Table 1.

**Figure 7:**
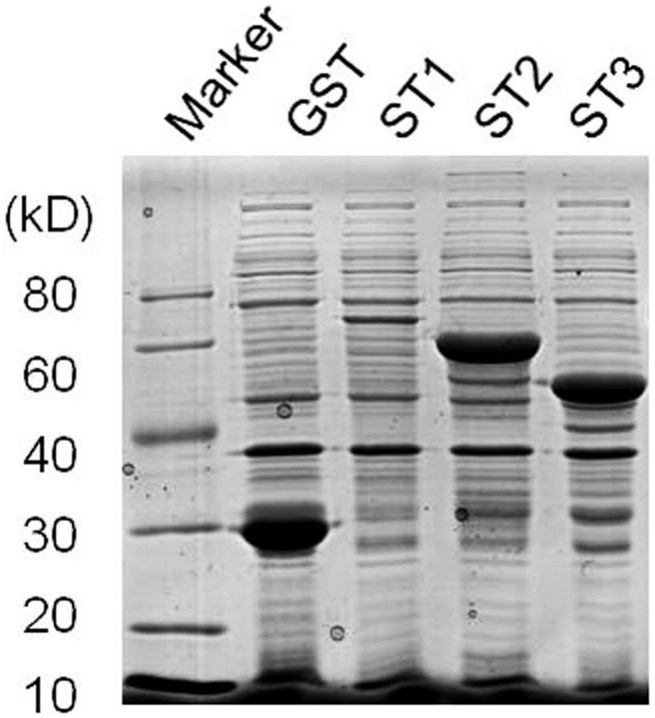
SDS-PAGE analysis of recombinant proteins. GST and GST-ST1, -ST2 and -ST3 fusions proteins were expressed in *E.coli*, extracted, and resuspended in loading buffer. The samples were separated on a 10% (w/v) SDS-polyacrylamide gel and visualized by Coomassie Brilliant Blue staining. Arrows indicate recombinant proteins.

Plant nucleosidases related to caffeine biosynthesis have not been reported previously. Tea transcriptome library [24] was analyzed for screening 7-mXR-specific nucleosidase. Based on sequence homology, six assembled contigs were selected from the published library as candidate genes: the singletons 7349, 46423, 53526, 109454, 115919, and 123579. The candidates were renamed ST1- ST6, respectively. The semi-quantitative RT-PCR results indicated that three of them, ST1, ST2, and ST3, showed a specific enhanced expression in young leaves, which corresponded to the suspected site of caffeine biosynthesis in tea (Fig. 6). These three proteins were expressed in *E. coli* (Fig. 7).

ST1, ST2, and ST3 were sub-cloned into yeast expression vectors and co-expressed with the two methyl-transferase genes described previously. Co-expression of these putative nucleosidases did not increase caffeine accumulation. These three genes were expressed in *E. coli* for functional analysis. Unfortunately, none of the purified proteins showed any activity towards 7-MX (data not shown).

### Identifying substrate binding amino acid residues of TCS1 and ICS1

**Figure 8:**
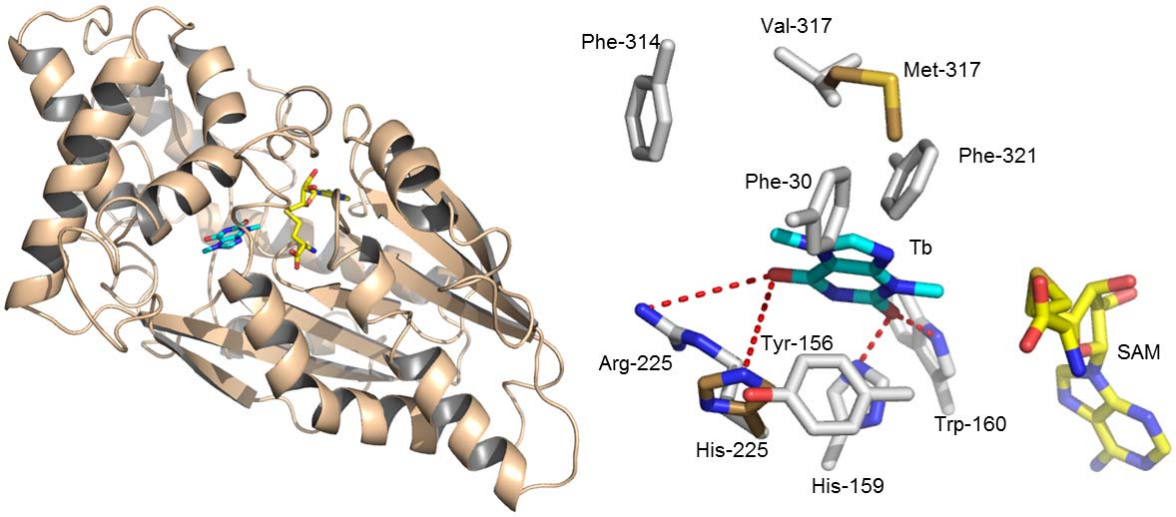
The tertiary model of TCS1 with theobromine (Tb) and SAM. (a) TCS1 model is represented as wheat cartoon; Tb and SAH (*S*-adenosine homocysteine) are represented as cyan and yellow sticks. (b) The close-up view of the Tb binding site of TCS1 wild type and mutants. Residues lining Tb binding sites of TCS1 wild type and mutants are colored in white and brown sticks, respectively.

The protein sequences of TCS1 from *Coffea arabica* and ICS1 from *Camellia irrawadiensis* were 56% homologous to 1,7-dimethylxanthine methyltransferase from *C. canephora*. It allowed us to undertake homology modeling and substrate docking analyses, based on the crystal structure of *C. canephora* DXMT (*PDB code: 2EFJ*). The TCS1 has high sequence identity (∼91%) with ICS1 and the tertiary structures of both proteins are similar. The homology models show that ICS1 and TCS1 exhibit obvious architectural changes in the putative active site in respect to the methyltransferase activity on theobromine. About 15 amino-acid residues of TCS1 constellated to constitute the binding pocket for the accommodated theobromine in the putative active site. Four amino acid residues (Arg225, Phe271, Ala272 and Val317) of TCS1 are distinct from ICS1, representing key substitutions changing the architecture of the active site (Fig. 8). These four distinct amino acid residues of TCS1 are replaced by His, Trp, Pro and Met in ICS1 respectively.

We attempted to mutate Arg225, Phe271, Ala272 and Val317 sites of TCS1 to their corresponding amino acid residues of ICS1 to alter its catalytic activity from caffeine to theobromine biosynthesis.

**Figure 9:**
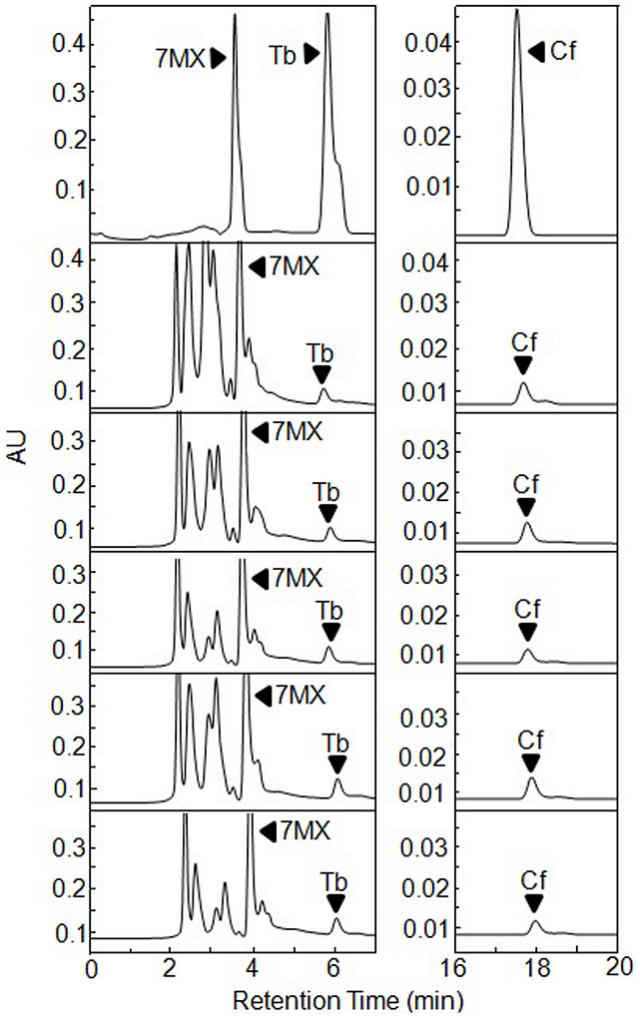
HPLC analysis after expression in *E. coli* of TCS1 or one of its putative active site mutants by using pGEX-4t-2 vector. (a) The trace shows the authentic standards run in parallel. (b) The trace shows the original TCS1 enzymatic reaction products. (c to f) show the HPLC analysis of the reaction products from the mutants TM1 to TM4. Black arrowheads indicate reaction products.

**Figure 10:**
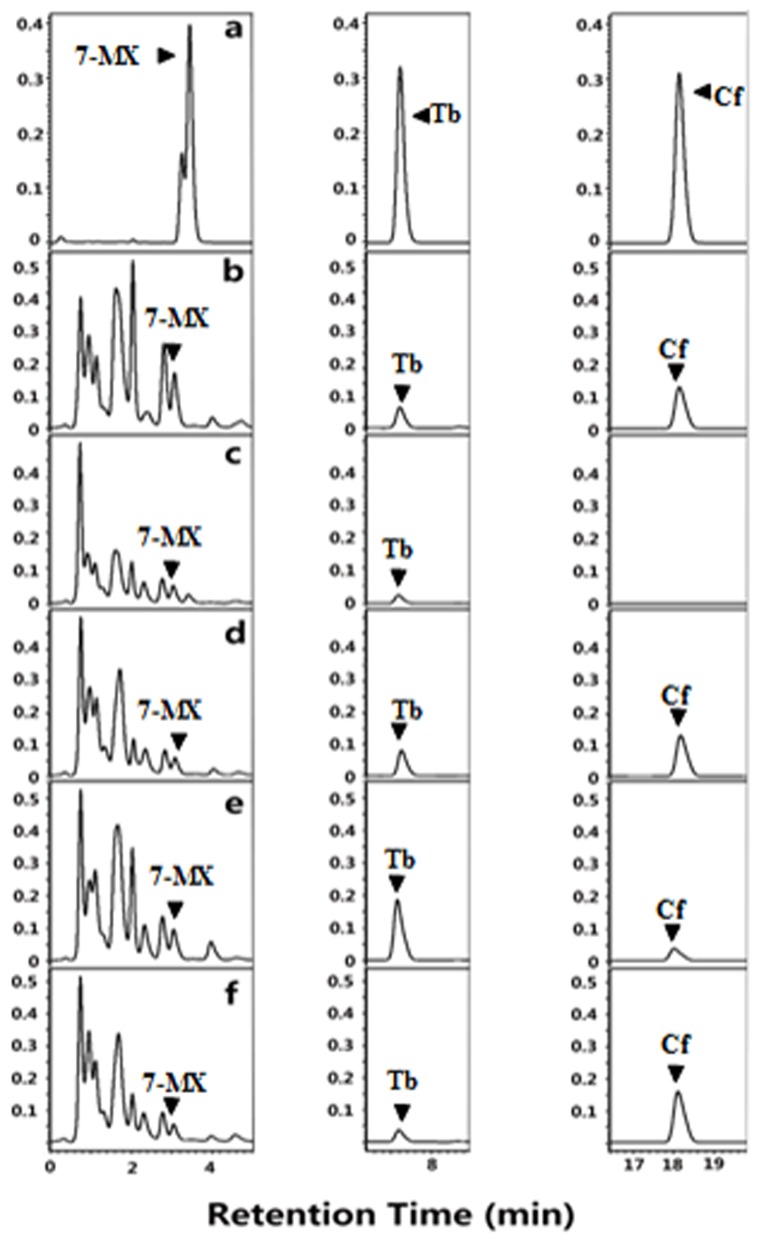
HPLC analysis after expression in *E. coli* of TCS1 or one of its putative active site mutants by using pMAL-c5X vector instead of pGEX-4t-2. (a) The trace shows the authentic standards run in parallel. (b) The trace shows the original TCS1 enzymatic reaction products. (c to f) show the HPLC analysis of the reaction products from the mutants TM1 to TM4. Black arrowheads indicate reaction products.

Four TCS1 mutants were expressed in *E. coli* and were tested for caffeine and theobromine synthesis. All of these four mutants of TCS1 produced same amount of caffeine compared with wild type (Figure 9). Only two mutants (Phe271Trp and Val317Met) produced slightly higher amount of theobromine (39.0 mg/l) compared to TCS1 wild type (35.0 mg/l) (Table 2). For the further improvement, a high efficient expression vector, pMAL-c5X was used. Together, the reaction condition was also modified with enough substrates provided. After the incubation, the products were determined by the same HPLC method (Figure 10). The sum yields of theobromine and caffeine in these two mutants (Phe271Trp and Val317Met) were 39.5 (30.54+8.98) and 33.3 (11.20+22.11) mg/l, respectively. It also showed these two mutants produced more theobromine and caffeine than the TCS1 wild type [31.9 (24.62+7.26) mg/l] (Table 3). The two independent experiment results suggested that these two sites (271 and 317) contributed to substrate specificity.

**Table 2:** Theobromine produced in enzymatic assays containing *E.coli* expressed TCS or TMX mutants by using pGEX-4t-2 and Rosetta (DE3).

Sample	Changed amino acid residue	Peak height (V)	Peak area (V*S)	Absolute ratio	Tb yields (mg/L)
TCS1	/	14939	174790	1.77	35.34
TM1	Arg225HIS	12343	149428	1.51	30.21
TM2	Val317Met	16544	195548	1.98	39.53
TM3	Phe271Trp	16035	191408	1.93	38.70
TM4	Ala272Pro	15389	179600	1.82	36.31
Tbstandard	/	8563	98928	1	20

**Table 3:** Theobromine and caffeine produced in enzymatic assays containing *E.coli* expressed TCS or TMX mutants by using pMAL-c5X vector instead of pGEX-4t-2.

Sample	Changedamino acid residue	Peak area (V*S)	Tb yields (mg/L)	Peak area(V*S)	Cf yields (mg/L)
TCS1	/	199301.45±13678.97	7.26±0.86	497210.95±36366	24.62±1.65
TM1	Arg225HIS	123181.85±23731.26	2.48±1.49	/	/
TM2	Val317Met	262060.6±51949.42	11.2±3.26	441727±39377.23	22.11±1.78
TM3	Phe271Trp	570311.38±33088.98	30.54±2.08	152291.65±14719.58	8.98±0.67
TM4	Ala272Pro	138970.65±40815.88	3.48±2.56	555705.5±32797.17	27.28±1.49
Tbstandard standard	/	454235	25	/	/
Cf standard		523461	25	/	/

Average values and s.d. (n = 4) are shown.

### Co-expression of CaXMT1 and TCS1 in *E. coli* system

**Figure 11:**
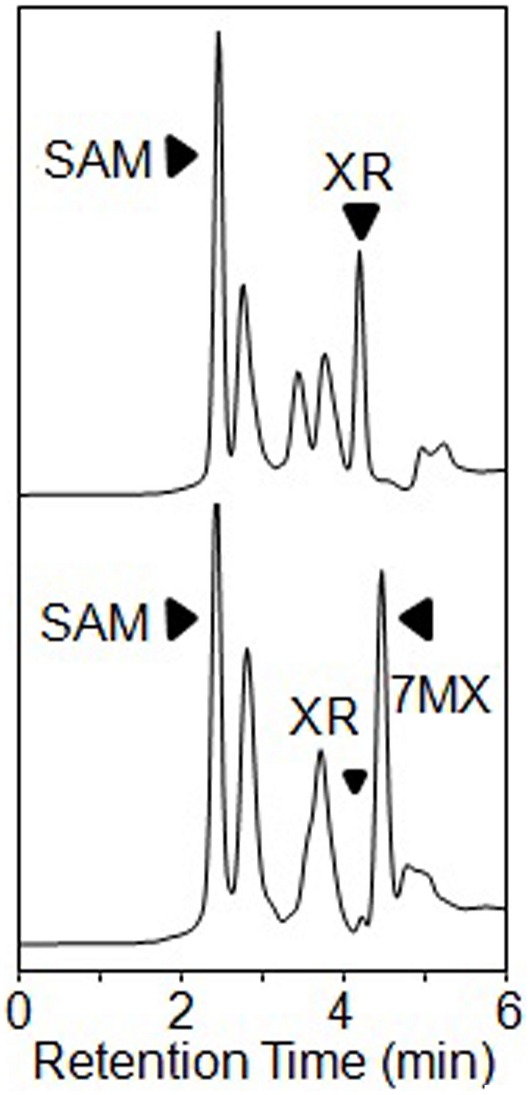
HPLC analysis after co-expression in *E. coli* of recombinant CaXMT1 andTCS1 by using pGEX-4t-2 vector. The top trace shows the negative control and the bottom trace shows the products of the sample enzymatic reaction. XR is substrate and SAM is added into the reaction as methyl donor.

**Figure 12:**
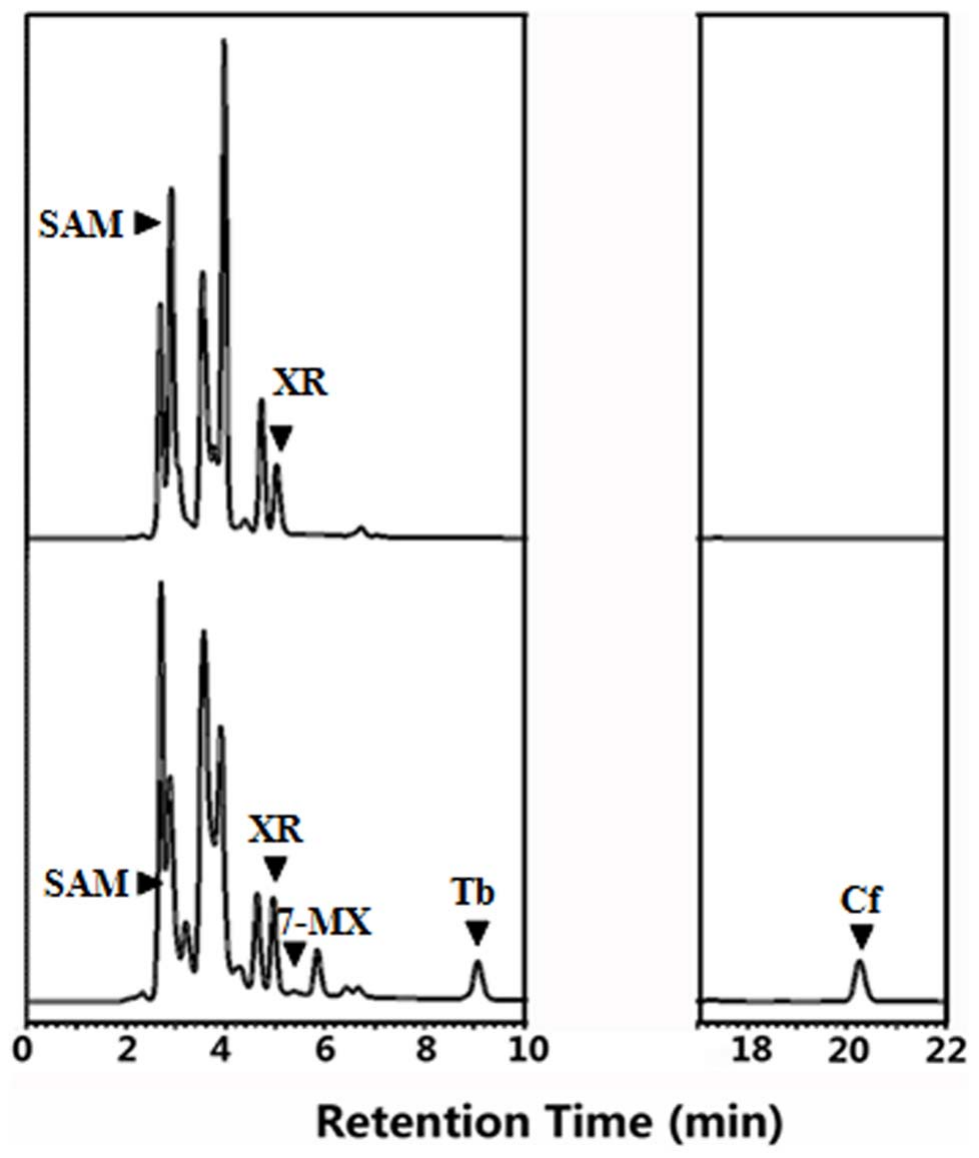
HPLC analysis after co-expression in *E. coli* of recombinant CaXMT1 andTCS1 by using pMAL-c5X vector instead of pGEX-4t-2. The top trace shows the negative control and the bottom trace shows the products of the sample enzymatic reaction. XR is substrate and SAM is added into the reaction as methyl donor.

We constructed the caffeine biosynthesis system in *E. coli* by co-expressing two genes. The two genes were cloned into pGEX-4t-2 expression vector and co-expressed in *E. coli*. However, unlike in the yeast system, only 7-MX was detected in the culture, and no detectable caffeine was found (Fig. 11). The functional analysis using the co-expressed cell extracts indicated that CaXMT1 activity could be detected, but the TCS1 activity was relatively low *in vivo*. Though the activity of both enzymes was detected *in vitro*, improvement of TCS1 activity is probably prerequisite for production of caffeine in bacterial culture. However, in order to improve the results, a new expression vector, pMAL-c5X (NewEnglandBiolabs, Beijing, China) was used instead of pGEX-4t-2. Figure 12 showed a low amount of caffeine was detected after the reaction system and incubation condition were optimized when CaXMT and TCS1 were co-expressed in *E. coli*.

## Discussion

Xanthosine methyltransferase (CaXMT1) from the leaf of *C. Arabica* was cloned. The enzymatic activity *in vitro* was detected by HPLC and confirmed by LC-MS, suggesting that CaXMT1 can directly catalyze the conversion from XR to 7-MX. The tea caffeine synthase (TCS1) was cloned from the leaf of *C. sinensis,* and it catalyzed the last two steps of caffeine synthesis, i.e. 7-MX→ Tb → Cf.

We co-expressed CaXMT1 and TCS1 in yeast and fed the substrate XR for production of caffeine. Successfully, the amount of caffeine was achieved to 0.38 mg/L. So far we know, this is the first report for production of caffeine in microbial systems. Xanthosine is a yeast metabolite. This endogenous xanthosine could also be used for caffeine synthesis. However, caffeine was not detected without exogenous xanthosine. This might be because the endogenous xanthosine in yeast is mainly used in primary metabolism and its amount was not enough to support caffeine biosynthesis.

We identified two potential bottlenecks to caffeine production in yeast based on reports that uridine nucleosidase (EC 3.2.2.3) could be inactivated by yeast protease A (EC 3.4.23.8) [33], which are the yeast endogenous nucleosidases with a broad substrate specificity [33]. It might encounter difficulty in removing the ribose residue from 7-methyl-xanthosine. In an attempt to increase caffeine production, we over-expressed a few putative tea nucleosidases in yeast.

Three nucleosidases from tea were identified from genomic library [24] and were selected based on their expression in leaves, where the majority of tea caffeine was produced [24]. Unfortunately, enzyme activity toward 7-mXR was not detected and co-expression of these enzymes with the two methyltransferases did not increase caffeine yield.

Yoneyama et al. [21] reported the isolation and characterization of *N*-methyltransferase from theobromine-accumulating *Camellia irrawadiensis* and *Camellia ptilophylla*. The recombinant forms of ICS1 and PCS1 exhibit only 3-*N*-methyltransferase activity [21], while TCS1 has both 3- and 1-*N-*methyltransferase activity. To further understand the mechanisms of substrate specificity, four TCS1 mutants (Arg_225_His, Val_317_Met, Phe_271_Trp, and Ala_272_Pro) were tested in yeast for increasing the production of caffeine. Only two of the mutants (Val_317_Met, Phe_271_Trp) showed slightly improved the production yields. Co-expression CaXMT1 and TCS1 *E. coli* did not produce any caffeine when pGEX-4t-2 was used. However, a low amount of caffeine was detected when CaXMT and TCS1 were coexpressed in *E. coli*, when one new expression vector, pMAL-c5X was used, and the reaction system and incubation condition were also improved at the same time.

This is the first report for fermentation production of caffeine by microbial culture and has provided important information for regulating caffeine biosynthesis in heterologous systems. Furthermore, site-directed mutagenesis of key caffeine synthetic enzymes gave insights for improvement of caffeine production. Structure-guided site directed saturation mutagenesis and co-expression of enzyme with transporters are in progress for further improvement of caffeine production.
